# Erie County Alms House

**Published:** 1849-04

**Authors:** 


*Erie County Alms House.*—Dr. J. B. Pride has been appointed Physician and Resident Superintendent of this Institution.

